# Effect of a Kinect-Based Exercise Game on Improving Executive Cognitive Performance in Community-Dwelling Elderly: Case Control Study

**DOI:** 10.2196/jmir.3108

**Published:** 2014-02-24

**Authors:** Hiroki Kayama, Kazuya Okamoto, Shu Nishiguchi, Minoru Yamada, Tomohiro Kuroda, Tomoki Aoyama

**Affiliations:** ^1^Human Health SciencesGraduate School of MedicineKyoto UniversityKyotoJapan; ^2^Division of Medical Information Technology and Administration PlanningKyoto University HospitalKyotoJapan

**Keywords:** fall prevention, cognitive function, dual-task, training, elderly

## Abstract

**Background:**

Decrease of dual-task (DT) ability is known to be one of the risk factors for falls. We developed a new game concept, Dual-Task Tai Chi (DTTC), using Microsoft’s motion-capture device Kinect, and demonstrated that the DTTC test can quantitatively evaluate various functions that are known risk factors for falling in elderly adults. Moreover, DT training has been attracting attention as a way to improve balance and DT ability. However, only a few studies have reported that it improves cognitive performance.

**Objective:**

The purpose of this study was to demonstrate whether or not a 12-week program of DTTC training would effectively improve cognitive functions.

**Methods:**

This study examined cognitive functions in community-dwelling older adults before and after 12 weeks of DTTC training (training group [TG]) or standardized training (control group [CG]). Primary end points were based on the difference in cognitive functions between the TG and the CG. Cognitive functions were evaluated using the trail-making test (part A and part B) and verbal fluency test.

**Results:**

A total of 41 elderly individuals (TG: n=26, CG: n=15) participated in this study and their cognitive functions were assessed before and after DTTC training. Significant differences were observed between the two groups with significant group × time interactions for the executive cognitive function measure, the delta-trail-making test (part B−part A; *F*_1,36_=4.94, *P*=.03; TG: pre mean 48.8 [SD 43.9], post mean 42.2 [SD 29.0]; CG: pre mean 49.5 [SD 51.8], post mean 64.9 [SD 54.7]).

**Conclusions:**

The results suggest that DTTC training is effective for improving executive cognitive functions.

**Trial Registration:**

Japan Medical Association Clinical Trial Registration Number: JMA-IIA00092; https://dbcentre3.jmacct.med.or.jp/jmactr/App/JMACTRS06/JMACTRS06.aspx?seqno=2682 (Archived by WebCite at http://www.webcitation.org/6NRtOkZFh).

##  Introduction

Cognitive impairment among elderly individuals is a serious issue in many countries. Many investigators have developed different cognitive function training methods as countermeasures to prevent cognitive impairment and have reported their effects [1]. Other investigators also have reported the effects of physical exercise training on cognitive functions among elderly individuals [2,3]. Additionally, Hillman et al and Silsupado have indicated that executive cognitive functions, which are related to the control of goal-oriented actions and adaptive behaviors, are strongly impaired by aging and respond positively to exercise training [4,5].

Recently, dual-task (DT) ability, or the performance of simultaneous motor and cognitive tasks, has been receiving considerable attention [6]. DT training is now recognized as a fall prevention tool that enhances physical functions among elderly people [7].

With a focus on DT, we developed a new concept called the Dual-Task Tai Chi (DTTC) test [8]. This system was developed using Kinect (Microsoft, Redmond, WA, USA), a motion-capture device, and demonstrated that the DTTC test quantitatively evaluates compound functions, including DT, balance, and cognitive abilities in elderly people [9]. In unpublished data, we found that DTTC training was useful not only to assess but also to improve balance and mobility among elderly people [10].

Several investigators have reported the effects of DT training on balance, mobility, walking, and DT ability [11,12]. However, only a few have reported that DT training improves cognitive performance. We reported that Trail-Walking Exercise, which is similar to Trail-Making Test (TMT) under DT condition, improved executive cognitive functions [13]. According to this, we expected that DTTC training would improve cognitive functions as well, especially executive functions. Therefore, the purpose of this study was to reveal that training with the DTTC device affects cognitive performance in elderly individuals.

## Methods

### Participants

Community-dwelling elderly subjects (n=48) participated in this study. The subjects were recruited through an advertisement in the local press. The following selection criteria were used: age ≥65 years, community dwelling, independent ambulation, willingness to participate in the measurement of physical fitness, and minimal hearing and vision impairment. Exclusion criteria were as follows: inability to complete the tasks because of reduced cognitive functions, evaluated by Rapid Dementia Screening Test [14] scored 8 or greater; severe cardiac, pulmonary, or musculoskeletal disorders; pathologies associated with increased risk of falls, such as Parkinson’s disease or stroke; osteoporosis; and psychotropic drug use. We obtained written informed consent from each participant. This study was approved (protocol approval E- 880) by the Ethical Review Board of Kyoto University Graduate School of Medicine, Kyoto, Japan.

### Device

The DTTC test [8-10] requires users to solve a number placement problem (Sudoku) by controlling a stick figure with the movement of their entire body. The user’s full-body motion is captured using Kinect and is translated into movements for the stick figure on a screen. The cognitive task is to fill in 3 boxes chosen at random from a 4 × 4 grid with digits ranging from 1 to 4. The user selects a digit using his or her right hand and left foot and points to a box with his or her left hand. In addition, the user must move his or her right hand to the left hand to fill the indicated box with the selected digit. As such, full-body motion, similar to Tai Chi Chuan movements, is required. We recorded the time taken to fill in all 3 boxes, as our evaluation index.

**Figure 1:**
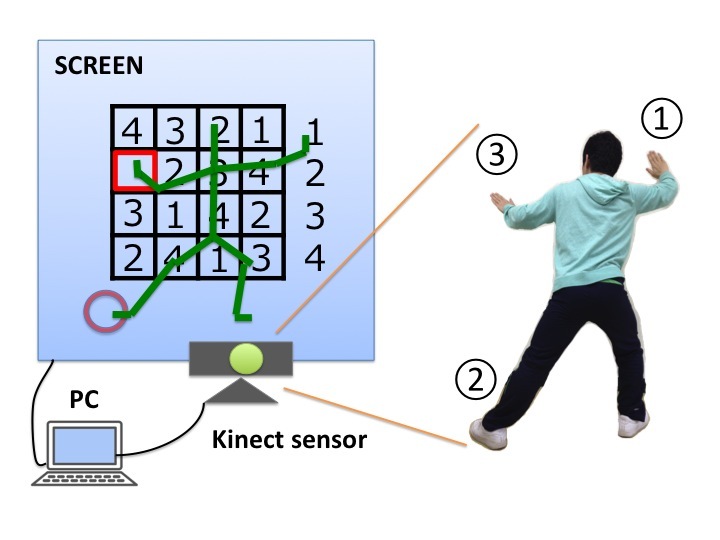
View of the Dual-Task Tai Chi (DTTC) test.

To begin with, the user stands 3 m in front of the Kinect sensor with his or her right foot in front of the sensor (Figure 1). The following instructions were provided:

1. Reach a digit you need to use with your right hand to fill a blank you want to answer.
2. Step 50 cm laterally, with your left leg, to grip the digit in your right hand.
3. Select the blank you want to answer with your left hand, and move your right hand to your left one.

### Intervention

All subjects participated in group training sessions lasting 75-80 minutes once a week for 12 weeks. The participants were divided into two training groups according to their participation in the exercise class: (1) the control group (CG), 75-minute standardized training, and (2) the training group (TG), 5-minute DTTC training, in addition to the standardized training [10].

The exercise classes were individualized for each group and were supervised by physical therapists. Each exercise class used a standardized format that included 15 minutes of moderate-intensity aerobic exercise, 15 minutes of progressive strength training, 10 minutes of flexibility and balance exercises, and 10 minutes of cool-down activities, followed by exercises known to improve muscle strength and balance [15,16]. In addition, the class included a 25-minute rhythmic stepping exercise involving cognitive ability, which is an exercise intended to improve DT ability [11], before cool-down.

The participants in the TG were additionally asked to solve DTTC problems and mirror-reversed DTTC problems, alternately, as many times possible in 5 minutes.

### Outcome Measures

All participants underwent evaluation upon entry into the study (pre-intervention) and at the end of the study (post-intervention), using the results of 2 cognitive performance tests.

Cognitive functions were evaluated using the trail-making test (TMT) [17] and verbal fluency test (VFT) [18-20]. The TMT is a well-established psychomotor test originally developed as part of the Army Individual Test Battery. The TMT has been widely used in clinical evaluations to assess deficits in executive cognitive functions. The test consists of 2 parts: part A is a visual-scanning task and part B is a measure of cognitive flexibility. For this analysis, we used a different score defined as delta-TMT, calculated as the difference between the times for each part (part B-part A). The delta-TMT score is used to control for the effect of motor speed on TMT performance and is considered a more accurate measure of executive functions than performance on part B alone [20,21].

The VFT has a letter fluency component and a category fluency component. Participants were asked to think of as many animal names as possible in 1 minute (category fluency). Verbal fluency is an evaluation of expressive language ability and executive functions. The score was the number of successful words (except for some proper nouns).

### Statistical Analysis

We compared baseline characteristics between the participants in each group to examine the comparability between the 2 groups using Student’s *t* test or the chi-square test. Repeated-measures, mixed-linear, two-way ANCOVA (analysis of covariance) was used to analyze the effect of exercise on outcome measurements while adjusting for each cognitive performance, at pre-intervention, as a covariate.

Data were entered and analyzed using the Statistical Package for the Social Sciences (Windows version 20.0, SPSS Inc., Chicago, IL, USA). For all analyses, *P*<.05 was considered statistically significant.

## Results

### Study Population

A total of 41 of the 48 selected subjects (85.4%) completed the study protocols and returned for their exit interviews and final testing (TG: n=26, CG: n=15). The participants’ baseline data did not differ significantly between the two groups. Thus, the groups were comparable and well matched with regard to their baseline characteristics.

### Adherence to the Study Protocols

During the 12-week intervention phase, 10 exercise sessions were scheduled and all took place. Excluding the 4 participants who dropped out, the TG subjects had an overall attendance rate of 82% and the CG subjects had an overall attendance rate of 81% over the 12 weeks. No health problems, including cardiovascular or musculoskeletal complications, occurred during training sessions or testing. Moreover, almost all participants seemed to have enjoyed the DTTC training. They shared many positive opinions after each session and seemed to look forward to playing DTTC once a week.

### Evaluation Outcomes

Pre- and post-intervention group statistics and group × time interactions are shown in Table 1. There was a significant difference between the groups regarding the changes (intervention to baseline) in delta-TMT. There were no significant differences among the other measures.

**Table 1:** Outcome measures by group at pre- and post-intervention.

Measures		Pre-intervention, mean (SD)	Post-intervention, mean (SD)	Group × Time *F* value	Degrees of freedom	*P* value
**VFT** ^a^						
	TG^b^	11.96 (3.55)	12.04 (3.26)	0.09	1,38	.76
	CG^c^	11.38 (4.21)	11.38 (4.07)			
**TMT-A** ^d^						
	TG	71.6 (23.8)	68.4 (19.9)	0.51	1,38	.48
	CG	82.2 (27.1)	70.0 (15.5)			
**TMT-B**						
	TG	120.3 (55.1)	110.4 (39.2)	2.73	1,38	.11
	CG	131.8 (62.6)	134.9 (61.1)			
**ΔTMT**						
	TG	48.8 (43.9)	42.2 (29.0)	4.94	1,36	.03
	CG	49.5 (51.8)	64.9 (54.7)			

^a^VFT: verbal fluency test

^b^TG: training group

^c^CG: control group

^d^TMT: trail-making test

## Discussion

### Principal Findings

The delta-TMT score was significantly improved after DTTC training. The results suggested that DTTC training was effective in improving executive cognitive functions. In our unpublished data, DTTC training was useful for improving balance ability and mobility among elderly people [10]. Thus, DTTC training has the capability of improving both physical and cognitive functions. Executive cognitive functions are closely related to DT performance and are good predictors of falling [20]. Thus, an improvement in executive functions, by DTTC training, has a positive impact on DT ability and the prevention of falling.

Conversely, the TMT-A and -B scores were not significantly improved. In the TMT-A, both groups improved similarly. On the other hand, in the TMT-B, only the TG had a tendency to improve the score, while CG showed little change. That is why the score of delta-TMT in CG increased and in TG decreased. The TMT-A and -B are used to assess visual scanning, cognitive flexibility, and executive functions [17]. The delta-TMT score is considered a more accurate measurement of executive functions [21,22]. That is, the results reﬂect specific improvement in executive cognitive functions. The reason is that DTTC training involves executive tasks that are the control of goal-oriented actions and adaptive behaviors. Additionally, VFT was also not significantly improved after DTTC training. It includes recalling tasks as their main elements; however, DTTC training does not involve recalling the task. This is considered the reason why the result of VFT was not significantly changed.

Previous studies reported that other “exergames,” based on Nintendo Wii Fit (Nintendo, Kyoto, Japan) or other devices, improved balance and leg muscle functions [23,24]. However, in these cases, the user needs control devices to capture motions and longer and more frequent exercise is required, compared with DTTC training, to obtain physical effects. DTTC overcomes these weaknesses and can offer comparable cognitive and physical benefits to users. Other previous studies have shown that a home-based dance device, using videogame technology, also improved the physical and cognitive parameters of fall risk in elderly people [25]. These home-based training tools are expected to increase the improvement of various functions and lead to fall prevention; our DTTC device is similar to these tools.

### Limitations

There are several limitations to this study. First, the intervention effects in this study were not due solely to the DTTC training. That means the rhythmic stepping exercise involving cognitive ability in both groups also improves cognitive ability. Second, participants in both groups may have had higher motivation and interest in health issues and fall risk minimization than the general elderly population. An investigation into the effects of DTTC training on functions in frail, elderly adults is necessary, in the future.

### Conclusions

In this study, the ANCOVA results of delta-TMT reveal that group × time interactions were statistically significant. They suggest that DTTC training is effective at improving executive cognitive functions in particular.

## Abbreviations

CG: control group
DT: dual task
DTTC: Dual-Task Tai Chi
TG: training group
TMT: trail-making test
VFT: verbal fluency test
